# Online Behavioral Addictions Among Adolescents Before and After the COVID-19 Pandemic

**DOI:** 10.7759/cureus.43231

**Published:** 2023-08-09

**Authors:** Berhan Akdağ, Arif Önder, Mehmet Emre Gül, Şevval Çınar Yorulmaz, Hilal Yazıcı Kopuz, Özge Gizli Çoban, Aslı Sürer Adanır

**Affiliations:** 1 Child and Adolescent Psychiatry, Silifke State Hospital, Mersin, TUR; 2 Child and Adolescent Psychiatry, Akdeniz University Faculty of Medicine, Antalya, TUR

**Keywords:** behavioral addiction, social media disorder, smartphone addiction, gaming disorder, covid-19

## Abstract

Background

The COVID-19 pandemic changed people's lives and created a "new normal." It threatened individuals' mental health owing to reduced physical activity and social interaction, excessive indoor time, financial hardship, and insecurity. Moreover, the risk of online behavioral addiction increased in the general population, particularly among adolescents. The present study examined the differences between the pre-and post-pandemic periods regarding online behavioral addictions in adolescents.

Methods

The pre-pandemic data were obtained from 175 adolescents (August 2019 to February 2020) (T1). An online survey was sent to these participants to obtain the post-pandemic data (March to September 2022) (T2). Seventy participants completed the online survey (response rate: 40%). The participants completed the Smartphone Addiction Scale (SAS), the Internet Gaming Disorder Scale 9-Short Form (IGDS9-SF), and the Social Media Disorder Scale-Short Form (SMDS-SF) both before and after the pandemic.

Results

Before the pandemic, females had significantly higher SMDS-SF scores compared to males (p = 0.005). On the other hand, males had higher IGDS9-SF scores than females before the pandemic (p<.001). Individuals with attention deficit hyperactivity disorder (ADHD) had higher IGDS9-SF scores before the pandemic than those with depressive disorders or other diagnoses (p = 0.004). However, the primary diagnosis was not related to pre-pandemic SAS and SMDS-SF scores. Lastly, there was no significant difference in IGDS9-SF (p = 0.151), SMDS-SF (p = 0.200), or SAS scores (p = 0.413) between pre-pandemic and post-pandemic scores.

Conclusion

Although the current study did not support this view, in emotionally challenging times, people may spend more time on online activities, which can lead to behavioral addiction. It is important for parents to monitor their children's online activities and provide guidance. More research is needed to compare online behavioral addictions before and after the pandemic.

## Introduction

The novel coronavirus disease (COVID-19) changed people's lives and created a "new normal." As in other countries, educational institutions, sports, and entertainment centers were closed in Turkey. The rise of digital technologies enabled many processes to be conducted online, leading to an increase in indoor activities. A new concept also entered the lives of students and parents: "remote learning."

The pandemic jeopardized individuals' bodily and mental health. Several factors have contributed to the increase in mental health problems: reduced physical activity and social interaction, excessive time spent indoors, financial hardship, uncertainty, insecurity, and the possibility of losing loved ones. One of the mental health problems that was exacerbated during the pandemic was addiction [1]. Indeed, a common view is that engaging in addictive behaviors aims to reduce distress [1]. In parallel, the risk of online behavioral addictions (e.g., problematic use of smartphones and social media and internet gaming disorder (IGD)) increased during the pandemic [2].

Online gaming is a popular leisure activity among the general population, especially among adolescents [3]. However, online gaming can become problematic when it interferes with psychosocial functioning. In this context, IGD has been included in the Diagnostic and Statistical Manual of Mental Disorders, Fifth Edition (DSM-5) as a condition warranting further study [4]. It has been accepted as a form of behavioral addiction (e.g., gambling disorder); in parallel, several symptoms have been identified for IGD, including excessive preoccupation with online gaming, tolerance, and withdrawal symptoms (e.g., irritability) [5]. Risk factors for IGD include impulsivity, attention deficit hyperactivity disorder (ADHD), anxiety disorders, and increased weekday game time [6]. During the pandemic, the closure of educational institutions and restrictions on outdoor time resulted in elevated online activities among adolescents [7]. Adolescents are particularly vulnerable because of their need for social connection and emotional support and are more likely to engage in online gaming activities to meet those needs [8]. As a result, an increase in the frequency of online gaming and the severity of IGD were observed among them during the pandemic [9].

Adolescents are the most digitally connected population worldwide [10]. Nevertheless, adolescents may also engage in addiction-like behaviors regarding social media use (SMU), such as the inability to control it or substitute it with other activities (e.g., hobbies) [10,11]. Similarly, excessive use of smartphones owing to the constant need to consult notifications and updates is problematic [12]. In the United States, 95% of adolescents had access to a smartphone, and 45% of them reported being online "almost constantly" [13]. Research among European adolescents also showed that intense SMU varied from 17.35% to 49.87%, whereas mixed problematic SMU ranged from 3.22% to 14.17% [14]. During the pandemic, virtual interactions and video chatting became popular, working from home and remote learning were recommended, and professional meetings were often conducted online [15]. Moreover, social media became essential for handling challenges and accessing basic needs such as communication, education, and entertainment [16]. As a result, while smartphones and SMU provide some benefits (e.g., staying in touch with relatives, especially if they live far away), the pandemic elevated the risk of addictive social media and smartphone use owing to spending more time online.

To summarize, adolescents spent more time online during the pandemic. Therefore, the present study aimed to compare the levels of smartphone addiction, social media addiction, and internet gaming disorder (IGD) in adolescents before and after the pandemic. We hypothesized that there would be an increase in the frequency and severity of these behavioral addictions among adolescents in the post-pandemic period compared to the pre-pandemic period.

## Materials and methods

Participants and procedure

The study sample consisted of adolescents followed up at the Akdeniz University School of Medicine Child and Adolescent Psychiatry Outpatient Clinic in Antalya, Turkey. Pre-pandemic data were collected between August 2019 and February 2020. A convenience sampling technique was used. The participants (n = 175) were adolescents followed up in the outpatient clinic. The mean age was 14.98 years (SD = 1.70, min-max: 12-18 years), and 90 (51.4%) participants were females. Of the participants, 24.7% had a diagnosis of ADHD, and 13.7% did not have any psychiatric diagnosis.

The pre-pandemic data (August 2019 to February 2020) (T1) were compared with those from the post-pandemic period (March 2022 to September 2022) (T2). An online survey was sent to the pre-pandemic participants (n = 175) to obtain post-pandemic data. Seventy participants completed the online survey (response rate: 40%).

Measures

The Smartphone Addiction Scale (SAS) is a 33-item instrument that measures smartphone addiction and consists of six dimensions: positive anticipation, daily-life disturbance, overuse, withdrawal, tolerance, and cyberspace-oriented relationships. Each item is scored from one (strongly disagree) to six (strongly agree); thus, the total score can range from 33 to 198. Higher scores reflect a higher smartphone addiction. During its development stages, the internal consistency coefficient was 0.97 [17]. The validity and reliability of the Turkish version were evaluated by Demirci et al. [18], and Cronbach’s alpha was 0.95.

The Internet Gaming Disorder Scale 9-Short Form (IGDS9-SF) is a brief measure adapted from the DSM-5 criteria describing internet gaming disorder. It aims to examine the severity of IGD and its detrimental effects in the last 12 months. The nine items are answered on a 5-point Likert scale (1 = never and 5 = very often), and higher scores indicate higher degrees of IGD. A score of 36 and above on the scale indicates a high risk of addiction [19]. The psychometric properties of the Turkish version were evaluated by Arıcak et al. [20], and Cronbach’s alpha was 0.82.

The Social Media Disorder Scale-Short Form (SMDS-SF) was designed to examine respondents’ experiences with social media [11]. The scale consists of nine dichotomous (yes or no) items that correspond to the diagnostic criteria for IGD in the DSM-5 [10]. The validity and reliability of the Turkish version were evaluated by Taş et al. [21], and Cronbach’s alpha was 0.76. A score of five or more on the scale indicates social media addiction.

Statistical analysis

Data analysis was performed using IBM Statistical Package for Social Sciences (SPSS) version 28.0 (IBM Corp., Armonk, NY, USA). The normality of the data was tested using the Kolmogorov-Smirnov test, skewness/kurtosis values, box plots, and histograms. The homogeneity of variances was analyzed with Levene’s test. Differences in scale scores between the two groups were tested using the Student’s t-test and Mann-Whitney-U test, and differences in the scale scores were tested using one-way analysis of variance (ANOVA) and the Kruskal-Wallis H test for multiple group comparisons. Spearman's correlation analysis examined the relationship between study variables. Pre- and post-pandemic SAS scores did not violate the assumption of normality. Thus, a paired-sample t-test was performed to compare the SAS scores. The Wilcoxon signed-rank test was performed on the comparisons of the IGDS9-SF and SMDS-SF scores owing to their non-normal distribution. 

## Results

The pre-pandemic characteristics of the participants are presented in Table 1.

**Table 1:** Pre-pandemic characteristics of the participants (n = 175) The total may not equal 100% due to missing data. M: mean; SD: standard deviation

	M (SD)
Age (years)	14.98 (1.70)
	n (%)
Gender	
Female	90 (51.4)
Male	85 (48.6)
Primary diagnosis	
Anxiety disorders	43 (24.6)
Attention deficit hyperactivity disorder	45 (24.7)
Depressive disorders	25 (14.3)
Others	38 (21.7)
No diagnosis	24 (13.7)
Education level (mother)	
Primary school or below	37 (21.2)
Secondary school	22 (12.6)
High school	52 (29.7)
University or above	35 (20.0)
Education level (father)	
Primary school or below	31 (17.7)
Secondary school	31 (17.7)
High school	40 (22.9)
University or above	38 (21.7)
Household income (Turkish Lira)	
<2000	32 (18.3)
2000-5000	94 (53.7)
>5000	44 (25.1)
Tobacco use	
Yes	33 (18.9)
No	142 (81.1)
Alcohol use	
Yes	16 (9.1)
No	159 (90.9)
Substance use	
Yes	2 (1.1)
No	173 (98.9)

Results of the pre-pandemic analyses

Table 2 summarizes the correlations for pre-pandemic variables.

**Table 2:** The correlations between study variables Spearman correlation analysis, **p<.01, ***p<.001 SAS: Smartphone Addiction Scale, IGDS9-SF: Internet Gaming Disorder Scale 9-Short Form, SMDS-SF: Social Media Disorder Scale-Short Form

	1	2	3
1. Pre-pandemic SAS	-		
2. Pre-pandemic IGDS9-SF	.360***	-	
3. Pre-pandemic SMDS-SF	.619***	.247**	-

Pre-pandemic SAS scores were positively related to pre-pandemic IGDS9-SF (ρ =.360, p<.001) and SMDS-SF scores (ρ =.619, p<.001). Similarly, pre-pandemic IGDS9-SF scores were positively associated with pre-pandemic SMDS-SF scores (ρ =.247, p = 0.001).

Females had significantly higher pre-pandemic SMDS-SF scores than males (U = 2893.5, p = 0.005). In contrast, pre-pandemic IGDS9-SF scores in male adolescents were higher than in female adolescents (U = 2239.0, p<.001). There is no difference between pre-pandemic SAS scores among males and females (t (173) = -.766, p = 0.445) (Table 3).

**Table 3:** The gender differences in pre-pandemic scale scores ^a^Student’s t-test, ^b^Mann Whitney-U test; IGDS9-SF: Internet Gaming Disorder Scale 9-Short Form, SAS: Smartphone Addiction Scale, SMDS-SF: Social Media Disorder Scale-Short Form

	Pre-pandemic SAS		Pre-pandemic IGDS9-SF	Pre-pandemic SMDS-SF
	M	SD	Median (Q1, Q3)	Median (Q1, Q3)
Males	91.62	34.73	18.0 (11.5, 24.0)	1.0 (0, 4.0)
Females	95.42	30.87	12.0 (9.0, 17.0)	3.0 (1.0, 5.0)
p-value	0.445^a^		<.001^b^	0.005^b^

**Figure 1:**
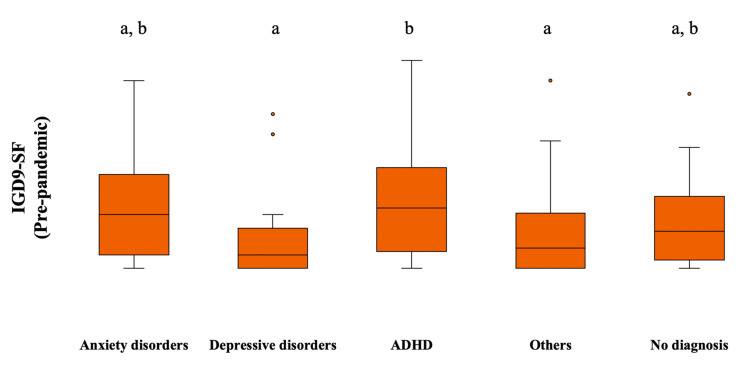
The relationship between pre-pandemic IGDS9-SF scores and primary diagnosis Different letters above bars indicate significant differences between groups (p < 0.05), while the same letters denote similar ranks between groups (p > 0.05) IGDS9-SF: Internet Gaming Disorder Scale 9-Short Form; ADHD: attention deficit hyperactivity disorder

Primary diagnosis was not associated with pre-pandemic SAS scores (F =.519, p = 0.722). Participants with ADHD had higher IGDS9-SF scores when compared with those with depressive disorders or other diagnoses (H = 15.30, p = 0.004). There was no difference between the other pairs (Figure 1).

On the other hand, the primary diagnosis was initially significantly associated with pre-pandemic SMDS-SF scores (H = 11.60, p = 0.021). However, after Bonferroni's adjustment, this relationship was no longer significant.

Comparison of pre-pandemic and post-pandemic scale scores

**Figure 2:**
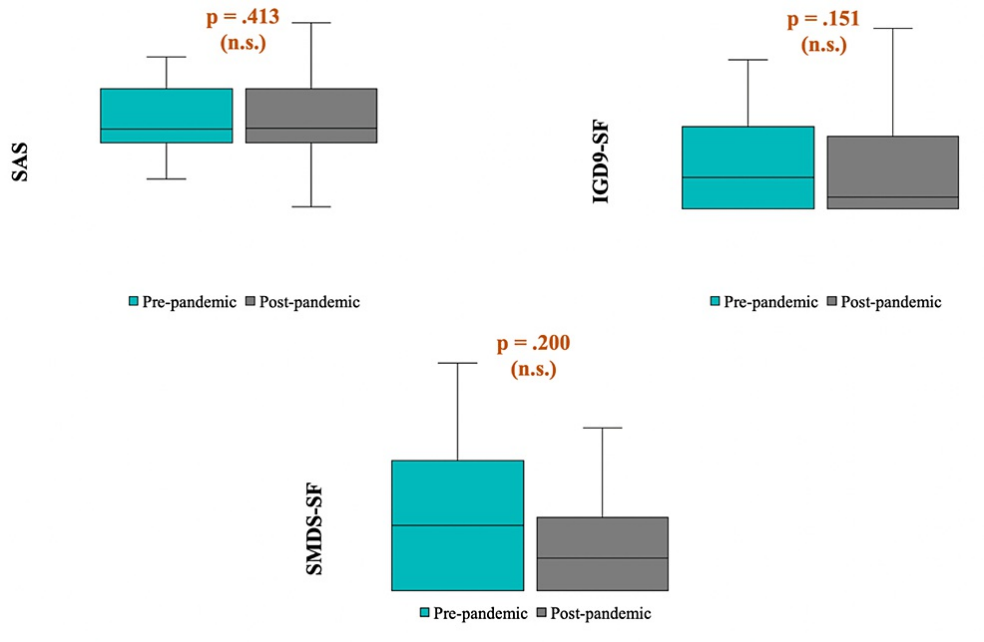
The differences between pre-and post-pandemic scale scores SAS: Smartphone Addiction Scale, IGDS9-SF: Internet Gaming Disorder Scale 9-Short Form, SMDS-SF: Social Media Disorder Scale-Short Form

Finally, a paired-sample t-test was performed to compare the pre-and post-pandemic scores of the SAS. No significant difference was observed in post-pandemic SAS scores (M = 93.71, SD = 31.84) compared with pre-pandemic SAS scores (M = 90.76, SD = 28.94); t (69) = -.823, p = 0.413). Similarly, no difference was observed between the pre-and post-pandemic scores of the IGDS9-SF (median (Q1, Q3): 13 (9, 21) versus 11 (9, 18), Z = -1.44, p = 0.151) and SMDS-SF (median (Q1, Q3): 2 (0, 4) versus 1 (0, 4), Z = -1.28, p = 0.200) scales (Figure 2).

## Discussion

The present study compared the levels of smartphone addiction, social media addiction, and internet gaming disorder (IGD) in adolescents before and after the pandemic. There was no significant difference in IGDS9-SF, SMDS-SF, and SAS scores between pre-pandemic and post-pandemic. Çakıroğlu et al. [22] reported that 54% of the participants spent more time with digital games during the pandemic than before the pandemic. Luo et al. [23] showed that the prevalence of social media addiction was 6.8% and that weekly SMU increased significantly during the pandemic (from 17.2 hours to 21.4 hours), and Teng et al. [9] found that IGD and video gaming increased among adolescents during the pandemic. Moreover, a systematic meta-analysis reported that the prevalence of behavioral addictions was 30.7% for smartphone addiction, 15.1% for social media addiction, 10.6% for internet addiction, and 5.3% for gaming addiction; the prevalence of gaming and social media addiction was also higher during lockdown periods [2]. In the present study, no significant differences were found between post- and pre-pandemic scale scores. There are possible explanations for these results. First, our sample size was small. Second, the sample characteristics might have contributed to these results. For instance, no participant was at high risk for internet gaming disorder (i.e., IGDS9-SF score above 36). Third, some participants (21.6%) continued their follow-up during the pandemic, which may explain the lack of increased frequency and severity of behavioral addictions. On the other hand, our results are similar to those of Sui et al. [24], who reported no significant difference between pre-and in-pandemic smartphone addiction or nomophobia (i.e., fear of being out of mobile phone contact) rates, although nearly all participants spent more time on a smartphone. Consequently, the COVID-19 pandemic seemed to increase the risk of behavioral addictions in the general population, especially adolescents, although the present study's findings do not support it.

The present study revealed higher pre-pandemic SMDS-SF scores in females than males. In contrast, pre-pandemic IGDS9-SF scores were higher in males. Durkee et al. [25] reported that the overall prevalence of problematic internet use was higher among males than females, with a significantly higher rate of online gaming in male adolescents and social networking in female adolescents. Chen et al. [26] also revealed that the prevalence of smartphone addiction among medical college students was 29.8% (30.3% in males and 29.3% in females); it was also associated with the use of gaming apps and social networking services in males and females, respectively. In summary, the broad view is that cell phone use is typically related to sociability (social networking), maintenance of interpersonal relationships, and communication among females. Males also use their time more practically and instrumentally (e.g., online gaming, texting, and voice conversation). Consequently, our findings are compatible with the available literature.

In this study, participants with ADHD reported higher IGDS9-SF scores than participants with depressive disorders or other conditions. Attention deficit hyperactivity disorder contributes to pathological gaming [27]. Indeed, brain abnormalities (e.g., gray matter volume and functional connectivity in the default mode network) in ADHD are associated with biological susceptibility to IGD and other addictive conditions [28,29]. Moreover, some neuropsychological characteristics, especially impulsivity, are associated with the comorbidity of IGD and ADHD [30]. Therefore, the findings of the present study are compatible with the available literature.

The present study is important because it addressed the differences between pre-and post-pandemic rates of smartphone addiction, social media addiction, and IGD in a sample of adolescents. In addition, it enriched the literature, as it is inconsistent with previous studies. However, some limitations should be emphasized. First, the sample size was small. Second, the findings cannot be generalized because nearly all participants had a psychiatric diagnosis. Third, factors potentially related to the study variables (e.g., mental state and personality traits) were not addressed. Fourthly, post-pandemic data were obtained online.

## Conclusions

The COVID-19 pandemic transformed people’s lives globally, and technology played a crucial role in this transformation. It served as a lifeline for many individuals to help them cope with distress. However, during emotionally challenging times, some individuals may spend more time on online activities, which can lead to behavioral addictions. It is essential to provide guidance to reduce the risk of such addictions. Parents should observe how much time adolescents spend on online activities (e.g., video games and SMU) and help them manage these activities.

## References

[1] Avena NM, Simkus J, Lewandowski A, Gold MS, Potenza MN (2021). Substance use disorders and behavioral addictions during the COVID-19 pandemic and COVID-19-related restrictions. Front Psychiatry.

[2] Alimoradi Z, Lotfi A, Lin CY, Griffiths MD, Pakpour AH (2022). Estimation of behavioral addiction prevalence during COVID-19 pandemic: a systematic review and meta-analysis. Curr Addict Rep.

[3] Tsui YY, Cheng C (2021). Internet gaming disorder, risky online behaviour, and mental health in Hong Kong adolescents: the beneficial role of psychological resilience. Front Psychiatry.

[4] American Psychiatric Association (2013). The Diagnostic and Statistical Manual of Mental Disorders, Fifth Edition.

[5] King DL, Delfabbro PH (2014). The cognitive psychology of Internet gaming disorder. Clin Psychol Rev.

[6] Rho MJ, Lee H, Lee TH, Cho H, Jung DJ, Kim DJ, Choi IY (2017). Risk factors for internet gaming disorder: psychological factors and internet gaming characteristics. Int J Environ Res Public Health.

[7] Terzioğlu MA, Uğurlu TT (2021). Perceived stress and nomophobia in medical faculty students during COVID-19 pandemic. Journal of Dependence.

[8] She R, Wong K, Lin J, Leung K, Zhang Y, Yang X (2021). How COVID-19 stress related to schooling and online learning affects adolescent depression and Internet gaming disorder: testing Conservation of Resources theory with sex difference. J Behav Addict.

[9] Teng Z, Pontes HM, Nie Q, Griffiths MD, Guo C (2021). Depression and anxiety symptoms associated with internet gaming disorder before and during the COVID-19 pandemic: a longitudinal study. J Behav Addict.

[10] Boer M, van den Eijnden RJ, Finkenauer C (2022). Cross-national validation of the social media disorder scale: findings from adolescents from 44 countries. Addiction.

[11] Van den Eijnden RJJM, Lemmens JS, Valkenburg PM (2016). The social media disorder scale. Comput Human Behav.

[12] Caponnetto P, Inguscio L, Valeri S, Maglia M, Polosa R, Lai C, Mazzoni G (2021). Smartphone addiction across the lifetime during Italian lockdown for COVID-19. J Addict Dis.

[13] Anderson M, Jiang J (2023). Teens, social media & technology 2018. https://www.pewresearch.org/internet/2018/05/31/teens-social-media-technology-2018/.

[14] Boer M, van den Eijnden RJ, Boniel-Nissim M (2020). Adolescents’ intense and problematic social media use and their well-being in 29 countries. J Adolesc Health.

[15] Gjoneska B, Potenza MN, Jones J (2022). Problematic use of the internet during the COVID-19 pandemic: good practices and mental health recommendations. Compr Psychiatry.

[16] Cleofas JV (2022). Social media disorder during community quarantine: a mixed methods study among rural young college students during the COVID-19 pandemic. Arch Psychiatr Nurs.

[17] Kwon M, Kim DJ, Cho H, Yang S (2013). The smartphone addiction scale: development and validation of a short version for adolescents. PLoS One.

[18] Demirci K, Orhan H, Demirdas A, Akpinar A, Sert H (2014). Validity and reliability of the Turkish version of the smartphone addiction scale in a younger population. Klinik Psikofarmakol Bulteni.

[19] Pontes HM, Griffiths MD (2015). Measuring DSM-5 internet gaming disorder: development and validation of a short psychometric scale. Comput Human Behav.

[20] Arıcak OT, Dinç M, Yay M, Griffiths MD (2018). Adapting the short form of the internet gaming disorder scale into Turkish: validity and reliability. Addicta Turk J Addict.

[21] Taş İ (2017). The study of validity and reliability of the social media addiction scale short form for adolescents [Article in Turkish]. Online Journal of Technology Addiction and Cyberbullying.

[22] Çakıroğlu S, Soylu N, Görmez V (2021). Re-evaluating the digital gaming profiles of children and adolescents during the COVID-19 pandemic: a comparative analysis comprising 2 years of pre-pandemic data. Addicta Turk J Addict.

[23] Luo T, Chen W, Liao Y (2021). Social media use in China before and during COVID-19: preliminary results from an online retrospective survey. J Psychiatr Res.

[24] Sui W, Sui A, Munn J, Irwin JD (2022). Comparing the prevalence of nomophobia and smartphone addiction among university students pre-COVID-19 and during COVID-19. J Am Coll Health.

[25] Durkee T, Kaess M, Carli V (2012). Prevalence of pathological internet use among adolescents in Europe: demographic and social factors. Addiction.

[26] Chen B, Liu F, Ding S, Ying X, Wang L, Wen Y (2017). Gender differences in factors associated with smartphone addiction: a cross-sectional study among medical college students. BMC Psychiatry.

[27] Lefler EK, Alacha HF, Vasko JM, Serrano JW, Looby A, Flory K, Hartung CM (2022). Sex differences in ADHD symptoms, problematic gaming, and impairment in college students. Curr Psychol.

[28] Lee D, Lee J, Lee JE, Jung YC (2017). Altered functional connectivity in default mode network in internet gaming disorder: influence of childhood ADHD. Prog Neuropsychopharmacol Biol Psychiatry.

[29] Lee D, Namkoong K, Lee J, Jung YC (2019). Preliminary evidence of altered gray matter volume in subjects with internet gaming disorder: associations with history of childhood attention-deficit/hyperactivity disorder symptoms. Brain Imaging Behav.

[30] Chen C, Dai S, Shi L, Shen Y, Ou J (2021). Associations between attention deficit/hyperactivity disorder and Internet gaming disorder symptoms mediated by depressive symptoms and hopelessness among college students. Neuropsychiatr Dis Treat.

